# Atrophy patterns of hippocampal subfields in T2DM patients with cognitive impairment

**DOI:** 10.1007/s12020-020-02249-w

**Published:** 2020-03-14

**Authors:** MengChun Li, LiLi Huang, Dan Yang, CaiMei Luo, RuoMeng Qin, Bing Zhang, Hui Zhao, Yun Xu

**Affiliations:** 1 Department of Neurology, Affiliated Drum Tower Hospital, Nanjing University Medical School, Nanjing, Jiangsu China; 2 Jiangsu Key Laboratory for Molecular Medicine, Nanjing University Medical School, Nanjing, China; 3 Nanjing Medicine Center For Neurological and Psychiatric Diseases, Nanjing, China; 4 Department of Radiology, Affiliated Drum Tower Hospital, Nanjing University Medical School, Nanjing, Jiangsu China

**Keywords:** Type 2 diabetes mellitus, Cognitive impairment, Hippocampal subfields, FreeSurfer, Magnetic resonance

## Abstract

**Purpose:**

To identify the volume changes of hippocampus subfields in T2DM patients with cognitive impairment and to determine how these atrophy patterns associate with impairments in different cognitive domain.

**Methods:**

A total of 117 individuals were recruited, including T2DM patients with cognitive impairment (T2DM-CI) (*n* = 34), T2DM patients without cognitive impairment (T2DM-non-CI) (*n* = 36) and normal controls (NC) (*n* = 47). All subjects went through a 3.0 T magnetic resonance (MR) scan and a neuropsychological assessment. Hippocampal subfield volumes were processed using the FreeSurfer 6.0.0 and compared among the three groups. Partial correlation analyses were used to estimate the relationship between cognitive function and hippocampal subfield volume, with age, sex, education, and eTIV (estimated total intracranial volume) as covariants.

**Results:**

The total hippocampal volume had a reduction trend among the three groups, and the significantly statistical difference only was found between T2DM-CI group and NC group. Regarding the hippocampal subfields, the volumes of left subiculum, left presubiculum, left fimbria, right CA1 and right molecular layer HP decreased significantly in the T2DM-CI group (*P* < 0.05/12). Partial correlation analyses showed that the volumes of the left subiculum, left fimbria, and left presubiculum were significantly related to executive function. The right hippocampal CA1 volume was significantly correlated with memory in the T2DM-CI group (*P* < 0.05). But in T2DM-non-CI group, the correlation between the left fimbria volume and the memory, the left subiculum volume and MoCA were different with the T2DM-CI group and NC group (*P* < 0.05).

**Conclusions:**

The smaller the volume of left presubiculum, the worse the executive function, and the atrophy of the right CA1 was related to memory impairment in T2DM-CI group. However the result was the opposite in T2DM-non-CI group. There might be a compensation mechanism of hippocampus of T2DM patients before cognitive impairment.

## Introduction

Type 2 diabetes mellitus (T2DM) has increased dramatically worldwide to become a public health burden in the past decades [1]. Large-scale epidemiological surveys have shown that one of the important complications of T2DM is cognitive impairment, including mild cognitive impairment (MCI), dementia, and Alzheimer’s disease (AD) [2, 3]. Also, T2DM contributes cerebellar vascular disease, white matter hyperintensities (WMHs), and cerebral atrophy in magnetic resonance imaging (MRI) imaging [4–6]. As we know, brain atrophy, particularly in the hippocampus, is associated with cognitive decline [7]. Moreover, the hippocampus is very sensitive to T2DM [8]. The loss of hippocampal neurons in diabetic encephalopathy is also found in rats [9].

The hippocampus is a C-shaped structure that spans the posterior-to-anterior in the centerpiece of medial temporal lobe [10]. It is not a uniform structure but is functionally heterogeneous, composed of different subfields, named as cornu ammonis (CA) subfields CA1–4, dentate gyrus (DG), fimbria, parasubiculum, presubiculum, and hippocampal tail. These subfields comprise the internal circuit and coordinate the function of the hippocampus [11]. For example, the CA2 subregion relates to social and emotional memory, the CA1 subregion is critical for autobiographical memory, and the hippocampal tail predicts depression status [12].

Atrophy of the hippocampus occurs before cognitive decline is apparent [13]. To date, very few studies have clarified the regional distribution of atrophy in the hippocampal subfields in patients with T2DM. How these internal structural changes are related to cognitive decline and how they influence early cognitive impairment in T2DM patients remains unclear. Therefore, it will be helpful to explore the mechanistic effects underlying T2DM-related cognitive impairment, especially as early as in the MCI stage.

Unlike most previous segmentation techniques of the hippocampus, 3 T high-resolution structural MRI provides the basic measurement of the hippocampal subfields regardless of the hippocampal surface changes [14]. FreeSurfer is a freely available voxel-based software package that provides extensive and automated neuroimaging analysis [15]. Compared with manual segmentation, FreeSurfer is highly efficient, accurate [16, 17], and has been successfully used to precisely segment the hippocampus [18–21]. A recent study in AD patients found that atrophy of the left subiculum correlated with cognitive disorders [19].

In this study, we aimed to investigate the morphology of hippocampal subfields in T2DM patients with and without cognitive impairment, and its relationship with cognitive performance in different subdomains. We hypothesized that T2DM patients with and without cognitive impairment would have heterogeneous hippocampal atrophy pattern, and the atrophy of hippocampal subfields mediated dysfunction in different cognitive domains. These efforts are important for understanding the pathophysiological changes occur during the development and progression of T2DM-related cognitive dysfunction.

## Method

### Participants

Participants were recruited from outpatients and inpatients of neurology department in the Affiliated Drum Tower Hospital of Nanjing University Medical School from January 2017 to February 2019. The T2DM participants were divided into the T2DM with cognitive impairment (T2DM-CI) group (*n* = 34) and the T2DM without cognitive impairment (T2DM-non-CI) group (*n* = 36) according to the neuropsychological test results. The diagnosis of T2DM was made according to the 2019 American Diabetes Association standards (fasting plasma glucose (FPG) ≥ 7.0 mmol/L, OGTT 2-h glucose ≥ 11.1 mmol/L, HbA1c ≥ 6.5%, or random plasma glucose ≥ 11.1 mmol/L in those who had clinical symptoms of hyperglycemia) [22], and the subjects without T1DM or taking diabetes medication were also defined as T2DM. The criteria for normal control (NC) group (*n* = 47) was cognitively normal and with no history of diabetes. All participants provided written informed consent that had been approved by the ethics committee of the Affiliated Drum Tower Hospital of Nanjing University Medical School.

The exclusion criteria for all participants included age <45 years; diabetes other than T2DM (e.g., T1DM, prediabetes); diabetes complications included (1) The acute metabolic complications such as diabetic ketoacidosis from exceptionally hyperglycemia, hypoglycemia coma [23]; (2) Microvascular complications such as eye disease seriously affected vision, renal insufficiency and diabetic neuropathy; (3) The major macrovascular complications such as cardiovascular disease and strokes) [24]; history of cerebral hemorrhage; cerebral infarction >15 mm on T2; Mini-Mental State Examination (MMSE) score < 20 (education years: 1–6) or reject to complete the neuropsychological test; other metabolic decompensation (e.g., hyperthyroidism); inability to give informed consent; illiteracy; family history of dementia; pregnancy; contraindications for MRI; brain trauma; major depression, alcoholism, psychiatric disorders or other mental disorders; or severe visual or hearing loss. All participants were right-handed.

### Clinical data

Data collected by self-report included demographics, health behaviors, and individual medical history (gender, age, education, duration of T2DM, hypertension, smoking and drinking history). Laboratory data collected by researchers included FPG, HbA1c, cholesterol (CHO), triglyceride (TG), low-density lipoprotein cholesterol (LDL-C), high-density lipoprotein cholesterol (HDL-C), blood urea nitrogen (BUN), creatinine (Cr), uric acid (UA), and eGFR.

### Neuropsychological testing

All subjects underwent a neuropsychological battery. The MMSE and Montreal Cognitive Assessment (MoCA) were adopted for evaluating global cognitive status. MMSE/MoCA both range 0–30 scores, and the higher scores represent better cognitive status. Our cognitive impairment group was diagnosed [25] when the MoCA score was ≤19 (education years: 1–6) or ≤24 (education years > 7). The Hamilton depression rating scale (HAMD) and Hamilton anxiety rating scale (HAMA) were adopted for assessment of emotional state. We defined severe depression as HAMD > 23 [26] and severe anxiety as HAMA > 28. The Auditory Verbal Learning Test (AVLT) and AVLT-delay recall (AVLT-DR) were used to assess memory, with higher scores representing better memory. The Trail Making Test (TMT) and Stroop Color Word Test (SCWT) were used to assess executive abilities [27–29]. The longer consuming time indicated worse executive performance. An experienced neuropsychologist conducted all the testing. The raw scores were transformed to *Z*-scores for each test [30, 31], which could be computed as follows:

$$Z = \frac{{x - \overline x }}{S},$$

where *x* is the raw scores, $\overline x$ is the mean of raw scores, *S* is standard deviation.

### MRI data acquisition

MRI data were obtained using a 3 T Philips Achieva Scanner (Philips, the Netherlands) equipped with an 8-channel head coil at Drum Tower Hospital. All participants underwent a 3-dimensional, high-resolution sagittal T1-weighted sequence scan, and the parameters were as follows: repetition time/echo time /inversion time = 9.8/4.6/900 ms, flip angle = 8°, field of view = 256 × 256 mm, matrix size = 256 × 256, and slice thickness = 1 mm. Fluid-attenuated inversion recovery (FLAIR) images and T1-weighted images were used for assessment of lacunar infarcts (LIs) and WMHs by Fazekas scores, which provided an indicator of the overall state of the WMH [32]. We defined LIs as spheroid/ovoid when FLAIR images showed a cerebrospinal fluid (CSF)-like hypointensity between 3 and 5 mm in diameter with a surrounding hyperintense rim [33]. In addition, T1-weighted images revealed when patients had hypointensity. We defined WMH as hyperintense on FLAIR [33]. All assessments were rated by experienced radiologists who were blinded to any clinical information.

### Data processing

T1-weighted images were processed by FreeSurfer version 6.0.0 using the default setting, which employed Bayesian inference with Markov random field priors [14] to obtain automated segmentations (http://surfer.nmr.mgh.harvard.edu/). Hippocampal subfield volumes and estimated total intracranial volume (eTIV) were obtained. The major recon stream for volume segmentation contains the following: within-subject motion correction, removal of nonbrain tissue using a hybrid watershed/surface deformation algorithm [34], affine registered to Talairach transformation, segmentation of the white matter, gray/white matter tessellation, automated topology correction, a probabilistic brain atlas registration which includes the subcortical and cortical structures (within hippocampal), and extraction of a label of each hippocampal subfield volume value that was estimated with a Bayesian statistical model. The specific procedures and algorithm have been published in previous research [14, 20, 35]. We visually inspected but did not edit any data. To reduce the influence of individual variation, the eTIV, which included gray matter (GM), WM and CSF volumes, was estimated and used as a covariate.

**Fig. 1:**
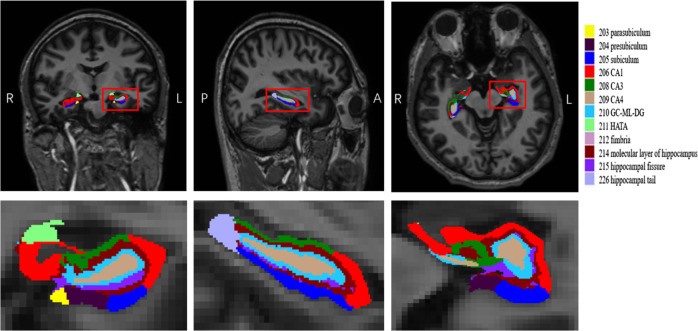
A simple of left hippocampal subfield automated segmentation

The hippocampus was automatically segmented into 12 subfields: hippocampal tail; subiculum; CA1; hippocampal fissure; presubiculum; parasubiculum; molecular layer of hippocampus; GC-ML-DG (granule cell and molecular layer of the DG); CA3; CA4; fimbria; and hippocampus amygdala transition area (HATA). We also obtained the whole volume of the bilateral hippocampi. The images from a normal subject are shown in Fig. 1 as an example.

### Statistical analysis

All data were analyzed using SPSS Statistics 20. Quantitative data were assessed for normality and shown as mean ± SD. Categorized data are expressed as frequencies (percentages) and compared using chi-square tests. The statistical threshold for significance was set as *P* value < 0.05 with Bonferroni correction (*P* < 0.05/12).

Differences in demographic, clinical, and neuropsychological data were assessed among the three groups by analysis of variance (ANOVA). Covariance analysis (ANCOVA) was applied to determine the hippocampal subregion volume differences among the three groups after controlling for age, sex, education, and eTIV.

To further investigate the associations between cognitive domains and hippocampal subregion volumes, the hippocampal subfields with *P* values < 0.05/12 were included in the partial correlation analyses. Age, sex, education, and eTIV were included as covariates.

## Result

### Demographic and clinical data

Both T2DM groups had higher FPG (*P* < 0.001) and HbA1c (*P* < 0.001) levels than the NC group, but there was no significant difference in FPG or HbA1c levels between the T2DM-CI and T2DM-non-CI groups. The T2DM-non-CI group had lower CHO and LDL-C levels than the NC group, while the T2DM-CI group had no significant differences in these two indexes compared to the NC and T2DM-non-CI groups. No significant differences were observed among the three groups in age, sex, education, body weight, BMI index, hypertension, smoking, and drinking history, WMH, LIs, eTIV, or laboratory examinations (TG, HDL-C, BUN, Cr, UA, eGFR). The demographic and clinical variables are shown in Table 1.

**Table 1:** Demographic and clinical data of all subjects

	NC (*n* = 47)	T2DM-non-CI (*n* = 36)	T2DM-CI (*n* = 34)	*F* or *χ*^2^ value	*P* value
Age (years, $\overline x$ ± s)	62.28 ± 8.66	64.11 ± 8.22	63.26 ± 8.12	0.496	0.610
Sex (male, %)	24 (51.1)	19 (52.8)	19 (55.9)	0.185	0.912
Education (years, $\overline x$ ± s)	11.79 ± 3.21	11.14 ± 4.16	10.62 ± 3.19	1.111	0.333
Duration (years, $\overline x$ ± s)	–	8.91 ± 6.25	7.78 ± 8.31	–	–
Body weight (kg, $\overline x$ ± s)	66.42 ± 12.19	64.13 ± 14.34	67.00 ± 10.52	0.186	0.831
BMI (kg/m^2^, $\overline x$ ± s)	24.25 ± 3.40	23.36 ± 5.01	25.24 ± 3.06	0.595	0.557
FPG (mmol/L, $\overline x$ ± s)	5.15 ± 0.7	6.16 ± 1.83	7.05 ± 2.79	10.234	<0.001^a,b^
HbA1c (%, $\overline x$ ± s)	5.55 ± 0.95	7.36 ± 2.78	6.87 ± 2.45	8.211	<0.001^a,b^
CHO (mmol/L, $\overline x$ ± s)	3.93 ± 1.25	3.26 ± 1.25	3.81 ± 1.16	3.312	0.040^a^
TG (mmol/L, $\overline x$ ± s)	1.36 ± 0.75	1.54 ± 0.89	1.75 ± 1.98	0.931	0.397
LDL-C (mmol/L, $\overline x$ ± s)	2.44 ± 0.68	1.97 ± 0.64	2.16 ± 0.77	4.515	0.013^a^
HDL-C (mmol/L, $\overline x$ ± s)	1.21 ± 0.36	1.06 ± 0.36	1.07 ± 0.34	2.143	0.122
BUN (mmol/L, $\overline x$ ± s)	5.17 ± 1.15	14.91 ± 56.04	5.73 ± 1.52	1.126	0.328
Cr (μmol/L, $\overline x$ ± s)	65.31 ± 15.58	63.67 ± 14.98	62.8 ± 17.45	0.249	0.780
UA (μmol/L, $\overline x$ ± s)	334.91 ± 84.15	331.83 ± 106.92	313.85 ± 77.72	0.573	0.566
eGFR(ml/min/1.73 m^2^, $\overline x$ ± s)	104.2 ± 18.13	106.5 ± 20.74	114.46 ± 31.97	1.798	0.171
Hypertension (*n*, %)	26 (55.3)	27 (75.0)	24 (70.6)	3.995	0.136
Smoking (*n*, %)	9 (19.1)	9 (25.0)	7 (21.9)	0.411	0.814
Drinking (*n*, %)	8 (17.0)	4 (11.1)	5 (15.6)	0.59	0.744
WMH (*n*, %)	0:4 (8.5)	0:2 (5.6)	0:2 (5.9)	5.213	0.517
	1:26 (55.3)	1:18 (50.0)	1:22 (64.7)		
	2:12 (25.5)	2:13 (36.1)	2:10 (29.4)		
	3:5 (10.6)	3:3 (8.3)	–		
LIs (*n*, %)	16 (34.0)	17 (47.2)	19 (55.9)	3.974	0.137
eTIV (cm^3^, $\overline x$ ± s)	1354810.8 ± 189364.1	1361174.12 ± 214428.56	1413738.08 ± 154738.46	1.083	0.342

*FPG* fasting plasma glucose, *CHO* cholesterol, *TG* triglyceride, *LDL-C* low-density lipoprotein cholesterol, *HDL-C* high-density lipoprotein cholesterol, *BUN* blood urea nitrogen, *Cr* creatinine, *UA* uric acid, *WMH* white matter hyperintensity, *LIs* lacunar infarcts, *eTIV* estimated total intracranial volume

*P* < 0.05 had statistical significance

^a^Compare NC group to T2DM-non-CI group

^b^Compare NC group to T2DM-CI group

### Neuropsychological assessment

Adjusted for age, sex, and education, the T2DM-CI group had worse performance on all neuropsychological tests than the NC group. There were lower scores in MMSE, MoCA, AVLT, and AVLT-DR; and longer consuming time in SCWTA, SCWTB, SCWTC, TMT-A, and TMT-B in the T2DM-CI group than that in the T2DM-non-CI group. In general, regarding global cognitive status measures, memory level and executive abilities were on a downward curve from the NC to T2DM-CI group. There was no difference in HAMD/HAMA among the three group. All raw scores and *Z*-scores of neuropsychological tests are shown in Table 2.

**Table 2:** Neuropsychological result of all group

	NC (*n* = 47)	T2DM-non-CI (*n* = 36)	T2DM-CI (*n* = 34)	*F* value	*P* value
MMSE score	28.79 ± 1.10	28.50 ± 1.36	27.24 ± 1.67	12.59	<0.001^a,b^
MoCA score	25.94 ± 1.73	26.03 ± 2.18	21.18 ± 2.48	80.959	<0.001^a,b^
HAMD	6.81 ± 5.10	4.72 ± 4.27	6.62 ± 5.28	1.955	0.146
HAMA	9.55 ± 7.29	6.78 ± 6.43	9.15 ± 6.61	1.736	0.181
Z-SCWTA consuming time	−0.242 ± 0.730	−0.176 ± 0.821	0.520 ± 1.289	5.977	0.003^a,b^
SCWTA consuming time	17.957 ± 5.086	18.417 ± 5.724	23.265 ± 8.983		
Z-SCWTB consuming time	−0.228 ± 0.840	−0.116 ± 0.918	0.438 ± 1.162	3.833	0.025^a,b^
SCWTB consuming time	20.702 ± 6.643	21.583 ± 7.26	25.971 ± 9.187		
Z-SCWTC consuming time	−0.175 ± 0.888	−0.071 ± 0.798	0.317 ± 1.261	2.197	0.116^a^
SCWTC consuming time	31.723 ± 11.517	33.083 ± 10.349	38.118 ± 16.361		
Z-TMT-A consuming time	−0.177 ± 1.103	−0.123 ± 0.784	0.376 ± 0.981	3.363	0.038^a,b^
TMT-A consuming time	54.085 ± 31.269	55.611 ± 22.226	69.765 ± 27.817		
Z-TMT-B consuming time	−0.234 ± 0.603	−0.173 ± 0.846	0.507 ± 1.369	6.426	0.002^a,b^
TMT-B consuming time	109.191 ± 80.664	117.306 ± 113.271	208.353 ± 183.259		
Z-AVLT score	0.270 ± 0.853	0.099 ± 1.073	−0.477 ± 0.965	5.532	0.005^a,b^
AVLT score	16.34 ± 3.246	15.694 ± 4.084	13.500 ± 3.67		
Z-AVLT-DR score	0.172 ± 0.915	0.223 ± 0.934	−0.473 ± 1.047	5.499	0.005^a,b^
AVLT-DR score	5.277 ± 2.018	5.389 ± 2.06	3.853 ± 2.311		

Adjusted age, sex, and education. *P* < 0.05 had statistical significance; Z meant converted raw scores to Z-scores; Z-SCWTmeant transformed SCWT consuming time to Z-SCWT consuming time; Z-TMT meant transformed TMT consuming time to Z-TMT consuming time; Z-AVLT meant transformed AVLT score to Z-AVLT score

*MMSE* Mini Mental State Examination, *MoCA* Montreal Cognitive Assessment, *HAMD* Hamilton depression rating scale; *HAMA* Hamilton anxiety rating scale, *SCWT* Stroop Color Word Test, TMT Trail Making Test, *AVLT* Auditory Verbal Learning Test, AVLT-DR Auditory Verbal Learning Test- dely remember

^a^Compare NC group to T2DM-CI group

^b^Compare T2DM-non-CI group to T2DM-CI group

### Analysis of hippocampal subfields

**Fig. 2:**
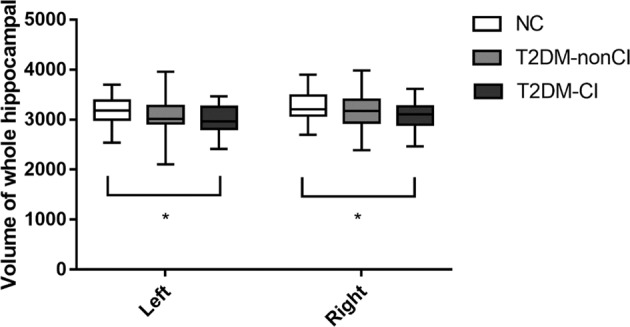
Comparison of bilateral whole hippocampal volume among three groups. Asterisk means *P* < 0.05

The bilateral total hippocampal volumes were compared among the three groups in Fig. 2. The hippocampus seemed to have a reducing trend across the three groups, but there is only significantly decreased in the T2DM-CI, not in the NC group (*p* < 0.05). There were no differences in total hippocampal volume between the NC and T2DM-non-CI group or between the two T2DM groups.

The bilateral hippocampi was divided into 24 subregions, the volumes of which are shown in Table 3. First, we compared volumes using ANCOVA after controlling for age, sex, education and eTIV. Significant differences were revealed in five subfields: the left subiculum (*F* = 6.09, *p* = 0.003), left presubiculum (*F* = 7.33, *p* = 0.001), left fimbria (*F* = 6.02, *p* = 0.003), right CA1 (*F* = 5.80, *p* = 0.004) and right molecular layer HP (*F* = 6.71, *p* = 0.002) among the three groups (*p* < 0.05/12). Then we performed post-hoc analyses with the least significant difference (LSD) in these five hippocampal subfields (Table 3). All five hippocampal subfields showed a significantly lower volume in the T2DM-CI group than in the NC group (*p* ≤ 0.001). But in T2DM-non-CI group, only the volume of the left presubiculum was smaller than that in the NC group (*p* = 0.007).

**Table 3:** Group comparison of hippocampal subfields volume

	Group			Anova		Post-hoc *p* value		
	NC (*n* = 47)	T2DM-non-CI (*n* = 36)	T2DM-CI (*n* = 34)	*F* value	*P* value	NC vs T2DM-non-CI	NC vs T2DM-CI	T2DM-non-CI vs T2DM-CI
Left hippocampal tail	524.01 ± 63.42	495.24 ± 81.08	508.93 ± 73.71	1.28	0.282	–	–	–
Left subiculum	405.45 ± 44.00	393.69 ± 58.62	375.97 ± 34.44	6.09	**0.003**	0.370	**0.001**	**0.017**
Left CA1	572.34 ± 65.54	563.44 ± 60.49	546.68 ± 57.69	2.40	0.095	–	–	–
Left hippocampal fissure	172.03 ± 27.36	167.81 ± 28.30	161.93 ± 18.76	1.96	0.146	–	–	–
Left presubiculum	306.18 ± 31.06	284.26 ± 45.11	281.93 ± 32.84	7.33	**0.001**	**0.007**	**<0.001**	0.373
Left parasubiculum	61.08 ± 10.84	55.68 ± 10.94	55.55 ± 9.571	4.15	0.018	–	–	–
Left molecular layer HP	513.69 ± 48.32	497.72 ± 54.92	484.42 ± 46.15	4.71	0.011	–	–	–
Left GC-ML-DG	264.81 ± 27.24	253.97 ± 27.16	250.23 ± 26.56	3.97	0.022	–	–	–
Left CA3	172.20 ± 25.50	167.23 ± 22.54	167.51 ± 23.24	0.51	0.601	–	–	–
Left CA4	225.53 ± 24.10	216.83 ± 23.26	214.27 ± 21.71	3.21	0.044	–	–	–
Left fimbria	79.11 ± 17.19	75.79 ± 20.08	68.35 ± 19.30	6.02	**0.003**	0.557	**0.001**	**0.01**
Left HATA	56.69 ± 7.61	52.29 ± 5.65	53.22 ± 7.30	4.53	0.013	–	–	–
Right hippocampal tail	554.60 ± 63.42	543.98 ± 71.96	534.93 ± 66.65	0.88	0.420	–	–	–
Right subiculum	412.63 ± 40.85	401.34 ± 56.18	385.93 ± 37.68	5.40	0.006	–	–	–
Right CA1	615.70 ± 72.39	600.51 ± 66.10	572.97 ± 54.65	5.80	**0.004**	0.323	**0.001**	**0.024**
Right hippocampal fissure	189.60 ± 27.42	185.64 ± 28.21	181.70 ± 22.06	1.34	0.265	–	–	–
Right presubiculum	288.00 ± 31.07	275.60 ± 41.52	279.12 ± 38.39	1.27	0.284	–	–	–
Right parasubiculum	55.04 ± 9.48	50.47 ± 8.99	53.50 ± 9.89	2.01	0.139	–	–	–
Right molecular layer HP	535.60 ± 51.79	520.45 ± 55.69	499.94 ± 43.70	6.71	**0.002**	0.271	**<0.001**	**0.017**
Right GC-ML-DG	269.41 ± 31.02	261.85 ± 29.25	254.65 ± 27.42	3.40	0.037	–	–	–
Right CA3	183.58 ± 29.26	178.33 ± 20.69	175.04 ± 26.70	1.16	0.319	–	–	–
Right CA4	227.60 ± 25.85	221.62 ± 25.68	216.96 ± 22.12	2.49	0.088	–	–	–
Right fimbria	69.78 ± 16.59	67.59 ± 20.75	66.65 ± 19.95	0.63	0.534	–	–	–
Right HATA	54.57 ± 7.93	53.43 ± 8.11	56.36 ± 8.66	0.82	0.443	–	–	–

Adjusted age, sex, education, and eTIV

Bold values indicates statistical significant *P* values

*P* < 0.05/12 had statistical significance

Post-hoc *p* < 0.05 had statistical significance

There were no statistically significant differences in the other subfields between the NC and T2DM-CI groups or the NC and T2DM-non-CI groups. The volume of the left subiculum (*p* = 0.017), left fimbria (*p* = 0.01), right CA1 (*p* = 0.024) and right molecular layer-HP (*p* = 0.017) was significant reduced in the T2DM-CI group compared with the T2DM-non-CI group. However, the left presubiculum volume (*p* = 0.373) was not significantly different between the two T2DM groups.

### Hippocampal subregion volume and cognitive function

**Fig. 3:**
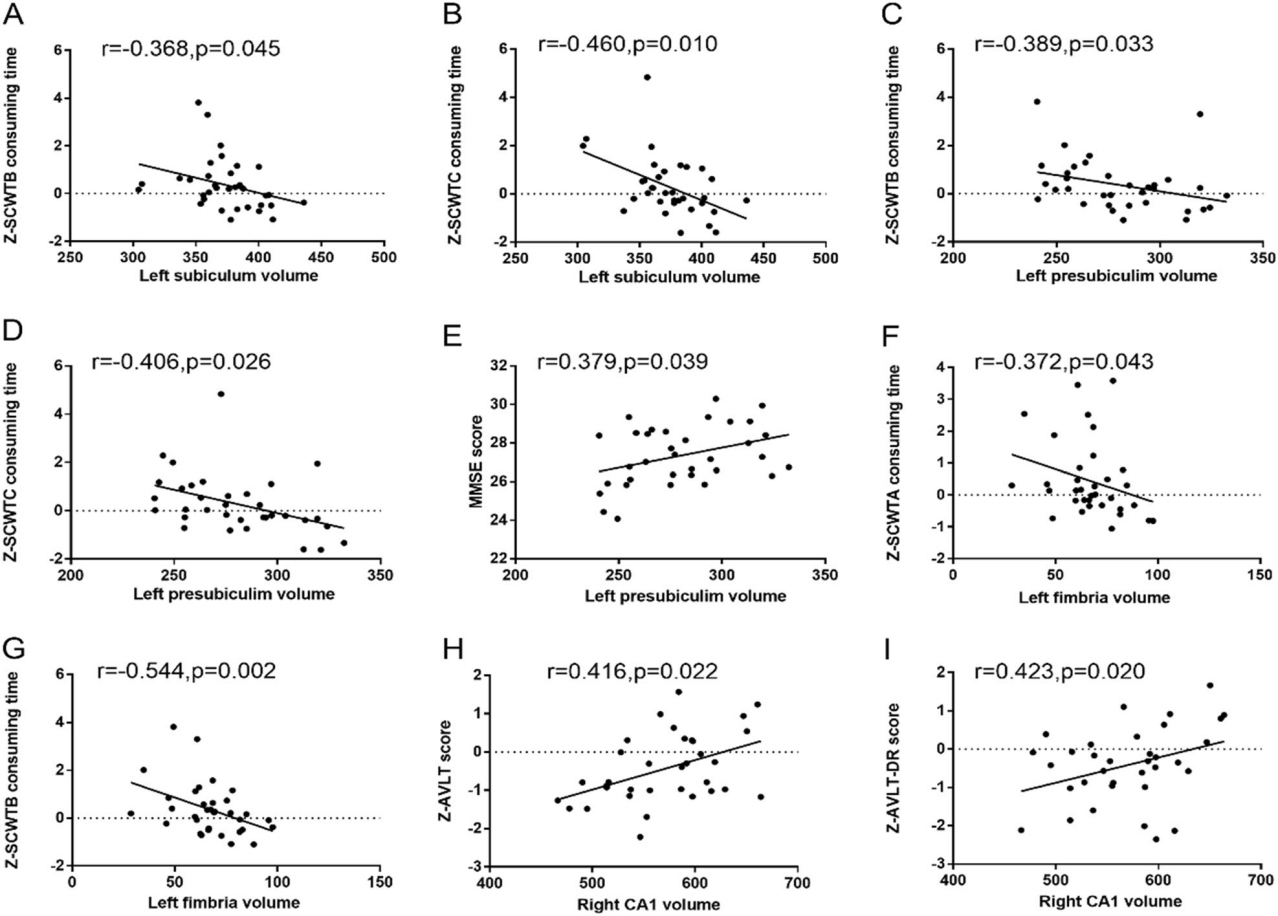
Partial correlation coefficient of cognitive function with hippocampal subregion volume in T2DM-CI patients. Adjusted age,sex and education and eTIV. *P* < 0.05 had statistical significance; MMSE mini mental state examination, MoCA montreal cognitive assessment, SCWT Stroop Color Word Test, TMT Trail Making Test, AVLT Auditory Verbal Learning Test, AVLT-DR Auditory Verbal Learning Test-dely remember

Table 4 and Fig. 3 presents the partial correlation analysis results between hippocampal subregion volumes and cognitive function in the T2DM-CI group, after adjusting for covariates (age, sex, education, and eTIV). Significant correlation was found between the left subiculum volume and executive function, as assessed by SCWTB consuming time (*r* = −0.368, *p* = 0.045) and SCWTC consuming time (*r* = −0.460, *p* = 0.010). The volume of the left fimbria was negatively correlated with executive function, as assessed by SCWTA consuming time (*r* = −0.372, *p* = 0.043) and SCWTB consuming time (*r* = −0.544, *p* = 0.002). The left presubiculum volume was found to correlate with SCWTB consuming time (*r* = −0.389, *p* = 0.033), SCWTC consuming time (*r* = −0.406, *p* = 0.026) and MMSE score (*r* = 0.379, *p* = 0.039). The right CA1 volume was correlated with memory, as assessed by AVLT score (*r* = 0.416, *p* = 0.022) and AVLT-DR score (*r* = 0.423, *p* = 0.020). There was no significant correlation between hippocampal subregion volumes and other cognitive function (MoCA, TMT-A consuming time, TMT-B consuming time).

**Table 4:** Partial correlation between cognitive function with hippocampal subregion volume in T2DM-CI patients

T2MD-CI group	left subiculum		left presubiculum		left fimbria		right CA1		right molecular layer_HP	
	*r*	*p*	*r*	*p*	*r*	*p*	*r*	*p*	*r*	*p*
Z-SCWTA consuming time	−0.292	0.117	−0.353	0.055	−0.372	**0.043**	0.201	0.286	0.225	0.232
Z-SCWTB consuming time	−0.368	**0.045**	−0.389	**0.033**	−0.544	**0.002**	0.176	0.351	0.030	0.876
Z-SCWTC consuming time	−0.460	**0.010**	−0.406	**0.026**	−0.236	0.209	0.008	0.967	−0.126	0.506
Z-TMT-A consuming time	−0.259	0.167	−0.348	0.059	−0.200	0.289	0.163	0.389	0.020	0.918
Z-TMT-B consuming time	−0.213	0.258	−0.120	0.529	−0.175	0.356	0.174	0.357	0.100	0.598
Z-AVLT score	0.165	0.382	−0.013	0.947	−0.002	0.992	0.416	**0.022**	0.345	0.062
Z-AVLT-DR score	0.225	0.232	−0.038	0.843	−0.163	0.388	0.423	**0.020**	0.302	0.105
MMSE	0.236	0.209	0.379	**0.039**	0.103	0.590	0.028	0.884	0.102	0.592
MoCA	−0.004	0.981	0.070	0.712	0.313	0.092	−0.048	0.800	−0.035	0.854
HAMD	−0.259	0.167	0.101	0.595	−0.121	0.525	0.023	0.902	0.126	0.505
HAMA	−0.104	0.586	0.145	0.444	−0.094	0.621	−0.058	0.759	0.092	0.627

Adjusted age, sex, education, and eTIV

*SCWT* Stroop Color Word Test, *TMT* Trail Making Test, *AVLT* Auditory Verbal Learning Test, *AVLT-DR* Auditory Verbal Learning Test-dely remember; *MMSE* Mini Mental State Examination, *MoCA* Montreal Cognitive Assessment, *HAMD* Hamilton depression rating scale, *HAMA* Hamilton anxiety rating scale

Bold values indicates statistical significant *P* values

*P* < 0.05 had statistical significance

**Fig. 4:**
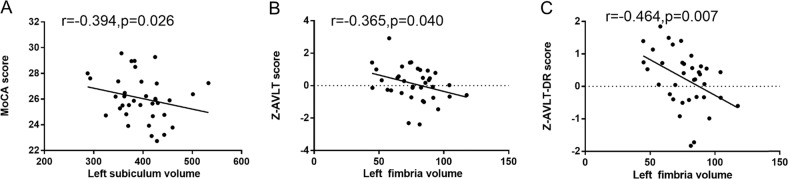
Partial correlation coefficient of cognitive function with hippocampal subregion volume in T2DM-non-CI group. Adjusted age,sex and education and eTIV. *P* < 0.05 had statistical significance, MoCA Montreal Cognitive Assessment; AVLT Auditory Verbal Learning Test, AVLT-DR Auditory Verbal Learning Test-dely remember

Table 5 and Fig. 4 shows the partial correlation analysis results in the T2DM-non-CI group after adjusting age, sex, education and eTIV. The volume of the left fimbria correlated with memory, as assessed by AVLT score (*r* = −0.365, *p* = 0.040) and AVLT-DR score (*r* = −0.464, *p* = 0.007). It also correlated with executive function as assessed by TMT-B consuming time (*r* = 0.413, *p* = 0.019), but when we excluded the clear outlier value, the significant was disappeared (*r* = 0.067, *p* = 0.719). The left subiculum volume was found to correlate with MoCA score (*r* = −0.394, *p* = 0.026).

**Table 5:** Partial correlation between cognitive function with hippocampal subregion volume in T2DM-non-CI patients

T2DM-non-CI group	left subiculum		left presubiculum		left fimbria		right CA1		right molecular layer_HP	
	*r*	*p*	*r*	*p*	*r*	*p*	*r*	*p*	*r*	*p*
Z-SCWTA consuming time	−0.292	0.105	−0.147	0.421	−0.025	0.890	−0.099	0.591	−0.192	0.292
Z-SCWTB consuming time	−0.112	0.543	0.111	0.544	0.150	0.413	−0.110	0.549	−0.048	0.795
Z-SCWTC consuming time	−0.231	0.203	−0.094	0.608	−0.049	0.790	−0.245	0.176	−0.172	0.345
Z-TMT-A consuming time	0.205	0.260	0.252	0.165	0.325	0.070	0.085	0.644	0.050	0.785
Z-TMT-B consuming time	0.099	0.590	0.100	0.588	0.413	**0.019**	0.272	0.132	0.128	0.485
Z-AVLT score	−0.273	0.130	−0.307	0.088	−0.365	**0.040**	0.015	0.937	0.023	0.901
Z-AVLT-DR score	−0.173	0.343	−0.057	0.756	−0.464	**0.007**	0.120	0.514	0.197	0.281
MMSE	−0.020	0.912	−0.125	0.496	−0.009	0.961	0.142	0.437	0.031	0.866
MoCA	−0.394	**0.026**	−0.150	0.411	−0.312	0.083	0.133	0.468	0.093	0.611
HAMD	−0.318	0.076	−0.146	0.426	−0.196	0.282	−0.270	0.135	−0.348	0.051
HAMA	−0.151	0.411	−0.085	0.642	−0.195	0.286	−0.322	0.073	−0.324	0.071

Adjusted age, sex, education, and eTIV

*SCWT* Stroop Color Word Test, *TMT* Trail Making Test, *AVLT* Auditory Verbal Learning Test, *AVLT-DR* Auditory Verbal Learning Test-dely remember, *MMSE* Mini Mental State Examination, *MoCA* Montreal Cognitive Assessment, *HAMD* Hamilton depression rating scale, *HAMA* Hamilton anxiety rating scale

Bold values indicates statistical significant *P* values

*P* < 0.05 had statistical significance

**Fig. 5:**
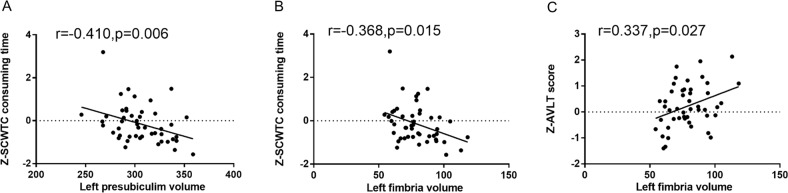
Partial correlation coefficient of cognitive function with hippocampal subregion volume in NC group. Adjusted age, sex, and education and eTIV. *P* < 0.05 had statistical significance; SCWT Stroop Color Word Test, AVLT Auditory Verbal Learning Test

Table 6 and Fig. 5 shows the partial correlation results between hippocampal subregion volumes and cognitive function in the NC group. There was a significant correlation between the left presubiculum volume and executive function, as assessed by SCWTC consuming time (*r* = −0.410, *p* = 0.006). The volume of the left fimbria was correlate with SCWTC consuming time (*r* = −0.368, *p* = 0.015) and AVLT score (*r* = 0.337, *p* = 0.027).

**Table 6:** Partial correlation between cognitive function with hippocampal subregion volume in NC patients

NC group	Left subiculum		Left presubiculum		Left fimbria		Right CA1		Right molecular layer_HP	
	*r*	*p*	*r*	*p*	*r*	*p*	*r*	*p*	*r*	*p*
Z-SCWTA consuming time	0.021	0.896	−0.106	0.497	−0.136	0.385	−0.175	0.261	−0.093	0.553
Z-SCWTB consuming time	−0.041	0.795	−0.250	0.106	−0.162	0.298	−0.236	0.127	−0.180	0.248
Z-SCWTC consuming time	−0.202	0.194	−0.410	**0.006**	−0.368	**0.015**	−0.063	0.686	−0.076	0.628
Z-TMT-A consuming time	0.097	0.538	0.119	0.446	−0.135	0.389	−0.007	0.963	−0.049	0.754
Z-TMT-B consuming time	0.176	0.258	0.133	0.395	−0.048	0.758	−0.010	0.948	−0.020	0.900
Z-AVLT score	0.042	0.789	0.237	0.126	0.337	**0.027**	0.096	0.540	0.103	0.510
Z-AVLT-DR score	0.120	0.444	0.211	0.173	0.232	0.134	0.282	0.067	0.279	0.070
MMSE	−0.058	0.712	−0.146	0.351	−0.180	0.247	0.045	0.775	0.062	0.692
MoCA	0.084	0.590	0.137	0.379	0.176	0.258	0.105	0.505	−0.033	0.833
HAMD	−0.189	0.226	−0.107	0.495	−0.083	0.597	0.015	0.925	−0.058	0.712
HAMA	−0.258	0.095	−0.106	0.498	0.077	0.625	−0.074	0.636	−0.146	0.351

Adjusted age, sex, education, and eTIV

*SCWT* Stroop Color Word Test, *TMT* Trail Making Test, *AVLT* Auditory Verbal Learning Test, *AVLT-DR* Auditory Verbal Learning Test-dely remember, *MMSE* Mini Mental State Examination, *MoCA* Montreal Cognitive Assessment, *HAMD* Hamilton depression rating scale, *HAMA* Hamiltonanxiety rating scale

*P* < 0.05 had statistical significance

### Laboratory data and hippocampal subfields volume

No significant correlations were found between any laboratory index and volume of the hippocampal subfields. In addition, the T2DM duration was not correlated with hippocampal volume.

## Discussion

In the present study, we analyzed the atrophy patterns of hippocampal subfields in T2DM patients with and without cognitive impairment. To the best of our knowledge, this is the first study focusing on the relationship between the atrophy of hippocampal subfields and T2DM-related cognitive domain impairment. Our data showed that the executive function and memory were the main impaired cognitive subdomain in T2DM patients. In T2DM-CI patients, the five atrophied hippocampal subregions were the left subiculum, left presubiculum, left fimbria, right CA1 and molecular layer-HP. In T2DM patients, the relationship between some subregions volume (the left presubiculum, the right CA1) and cognitive performance was seemed to stable, but in some subregions (the left fimbria, the left subiculum) the relationship were changeable when T2DM patients with or without cognitive impairment. Finally, the atrophy of these particular subfields of the hippocampus was related to executive function and/or memory impairments in T2DM patients.

The neuroimaging studies of T2DM patients have shown inconsistent results regarding WMH and infarctions [7, 36, 37]. In our study, there was no difference in WMH or LIs among all groups. We found a smaller whole hippocampal volume in the T2DM-CI patients than in the NC subjects, which was similar to a previous report [38]. But in this study, we used more detailed hippocampal subregion structure and more specialized cognitive subdomains tests in order to explore the morphological changes of T2DM-realted cognitive impairment. However, we did not observe hippocampal atrophy in the T2DM-non-CI group (Fig. 2). These results were slightly different from previously reported results. For example, in elderly participants T2DM patients had more atrophy of the hippocampus than control [39]. The reason might be the differences in the subjects. In our research, T2DM patients were divided into two groups according to the presence of cognitive impairment. However, most previous studies [38] focused only on T2DM patients as a group and ignored the influence of cognitive differences, This study focus on T2DM-realted cognitive impairment. These results demonstrated that the atrophy of the main region of memory formation still played a crucial part in T2DM-related cognitive impairment.

But as we mentioned above, the hippocampus consists of more than ten subfields that related with different cognitive function. Previous studies in T2DM-related cognitive impairment, focused on the change of the whole hippocampus, little is known about the changes in hippocampal subfields. So, in our work, the T2DM patients were divided in two groups according to whether there was cognitive decline or not, and the bilateral hippocampus were automated segmented into 24 subfields using FreeSurfer. The more detailed grouping would help us to precisely identify smaller lesions changes related to early cognitive impairments in T2DM patients, compared with previous research [40].

In this study, among the three groups, the atrophy of the left subiculum, presubiculum and fimbria, and the atrophy of the right CA1 and molecular layer-HP in the T2DM-CI group were the most significant. Previous studies have suggested that a smaller volume of the subiculum and CA1 might be responsible for memory impairments in T2DM patients [38]. The subiculum was crucial for cognitive functions such as communication, behavioral performance and exploratory behavior [41]. Anatomical and physiological studies proved that the projection pathway from the subiculum interacts with the CA1 to regulate hippocampal circuit activity and learning and memory [42]. Our results were supported by previous studies, which reported that the subiculum and CA1 related with cognition in T2DM rat and human [38, 43, 44]. A structural imaging study in patients with subjective cognitive decline found volume reductions in the presubiculum, molecular layer and fimbria [19]. Another study in a large population-based cohort reported smaller volumes of the hippocampal fimbria, presubiculum and subiculum that was related to a risk of dementia [45]. Both basic research and clinical research has confirmed the roles of these hippocampal subfields in learning and memory. Our findings about the hippocampal subfield volume reductions in the T2DM-CI group were consistent with these findings and reflected an earlier stage of cognitive disorder in T2DM.

The cognitive impairments of patients with T2DM might involve every domain [46, 47]. However, according to a previous paper, the most common domains that were affected were executive functions and memory [40, 48]. Our results in T2DM-CI patients revealed that the volume changes of the left presubiculum were correlated with the MMSE scores and the atrophy of the left subiculum, the left presubiculum and the left fimbria were related to executive function, while the atrophy of the right CA1 was related to memory. This coincided with the study of Evans et al., who found that the decreased volume of the hippocampal subiculum, presubiculum and fimbria was strongly associated with poor performance on executive function, not memory [45]. However, no similar result has been found in T2DM patients according to our knowledge, further study is needed.

Then we did partial correlation analysis between hippocampal subregion volumes and cognitive function in NC group and T2DM-non-CI group. It revealed that the volume of the left presubiculum and fimbria were related to executive abilities in general population. Interestingly, this study indicated there was positive relationship between the atrophy of volume in the left subiculum/fimbria and the executive function decline in T2DM-CI group. However, in T2DM-non-CI group, although no cognitive decline, the neuropsychological test score was negatively correlated with the volume of the left subiculum/fimbria. There might be a structural compensation of hippocampal subfields in T2DM patients before MCI. Maybe there also would be a complex functional regulation mechanism in very early changes of cognitive in T2DM patients. To answer these questions, further basic experiment and a larger research should focus on it.

We did not find significant correlation between the level of blood glucose or duration of disease and cognitive impairment. But the hippocampal volume in the T2DM-CI group was significantly reduced compared with the NC group. The atrophy of the hippocampus might occur earlier than the onset of cognitive symptoms, which could also indicate that the brain was influenced by high blood glucose. In a recent study examining associations in older adults between diabetes with incident cognitive impairment, poor glycemic control and longer diabetes duration were associated with worse cognitive outcomes over a median follow-up of 5 years [49]. However, a recent systematic review thought that the effect of T2DM treatment on the development of cognitive impairment and dementia still was a complex issue [50]. The relationship between glycemic control and cognitive impairment requires a larger sample and longitudinal study.

Our current study had several limitations. The first was the relatively small patient sample size, which might have limited the statistical power of this study. A larger dataset of participants is necessary to make the results more robust. Second, we considered the values of the clinical indicators and the duration of T2DM. However, the medications of the T2DM patients were not completely identical, so confounding effects of the medications might have existed and affected cognitive function. Finally, this was only cross-sectional data. Although the result could help understand the progression of cognitive impairments in T2DM patients, longitudinal follow-up studies of the same cohort should be conducted to identify early imaging markers for disease transformation and prediction.

In summary, the present study provides evidence of hippocampal subfield volume changes in T2DM patients with cognitive decline. Moreover, the changes in specific regions of the hippocampus were associated with different cognitive domains, such as executive abilities and memory. These specific associations will help to identify early cognitive decline in T2DM, to understand the underlying biological mechanism and contribute to interventions for T2DM-associated cognitive impairments.
